# Prevention of pin site infection in external fixation: a review of the literature

**DOI:** 10.1007/s11751-016-0256-4

**Published:** 2016-05-12

**Authors:** Nikolas H. Kazmers, Austin T. Fragomen, S. Robert Rozbruch

**Affiliations:** 1 Department of Orthopaedic Surgery, University of Pennsylvania, 3737 Market Street, 6th Floor, Philadelphia, PA 19104 USA; 2 Limb Lengthening and Complex Reconstruction Service, Hospital for Special Surgery, Weill Medical College of Cornell University, 535 East 70th Street, New York, NY 10021 USA

**Keywords:** Pin site, Pin tract/track, Infection, Prevention, External fixation, Limb lengthening

## Abstract

Pin site infections are a common complication of external fixation that places a significant burden on the patient and healthcare system. Such infections increase the number of clinic visits required during a patient’s course of treatment, can result in the need for additional treatment including antibiotics and surgery, and most importantly can compromise patient outcomes should osteomyelitis or instability result from pin loosening or need for pin or complete construct removal. Factors that may influence the development of pin site infections include patient-specific risk factors, surgical technique, pin design characteristics, use of prophylactic antibiotics, and the post-operative pin care protocol including cleansing, dressing changes, and showering. Despite numerous studies that work to derive evidence-based recommendations for prevention of pin site infections, substantial controversy exists in regard to the optimal protocol. This review comprehensively evaluates the current literature to provide an overview of factors that may influence the incidence of pin site infections in patients undergoing treatment with external fixators, and concludes with a description of the preferred surgical and post-operative pin site protocols employed by the senior authors (ATF and SRR).

## Introduction

Pin site infections are common orthopaedic problems that may arise from percutaneous pins or wires [1–62]. These complicate the use of skeletal traction pins, percutaneous fracture pinning, and external fixation; the optimal methods to prevent and treat them are controversial [4, 6, 10, 17, 18, 21, 24, 25, 30, 33, 35–39, 51–53, 55, 57, 58, 63, 64]. Pin sites are susceptible to infection because the skin barrier has been disrupted. One series reports pin site infections to be the most common complication of external fixation, occurring in up to 100 % of the study group [33, 52].

Most pin site infections are treatable with improved wound care and a short course of oral antibiotics [25]. Deep tissue infections and osteomyelitis may occur in up to 4 % of cases [19, 36], which are serious complications. Pin loosening, increased pain, use of pain medications, and delayed mobilization may follow [55]. Pin removal may be required in severe cases that fail to respond to antibiotic treatment [14, 36]. Due to a high incidence of pin site infections in patients undergoing external fixation and the morbidity and costs associated with the sequelae, clinicians need to educate patients to recognize the signs and symptoms of pin site infections in order to initiate treatment promptly. Orthopaedic surgeons need to recognize a spectrum of pin site infections, to identify patients who are at increased risk, and to anticipate the more advanced complications that may arise from a simple pin site infection. The optimal methods that should be used to prevent pin site infections are debated, and this reflects the many hospitals which utilize dissimilar post-operative pin site care protocols [21, 36]. A critique of the current evidence is presented.

### Definition and classification of pin site infections

There is no accepted definition of pin site infection universally [25]. Comparisons of results by different studies, which range from 0 to 100 %, are difficult. This large discrepancy reflects variations in what is considered a true pin infection, the study duration, the external fixator application technique, patient population, and the pin site care protocol used [33]. A lack of a universal definition and reporting of infection rates (per patient versus per individual pin site) pose challenges to conducting a systematic review.

### An attempt to differentiate three levels of skin reaction to percutaneous pins has been reported: [24]

1. *Pin site reaction*: Represents normal/physiologic changes in skin colour, skin warmth, and pin site drainage and resolves within 72 h.
2. *Pin site colonization*: Includes Erythema, warmth, drainage, possible pain, and positive culture.
3. *Pin site infection*: Includes all of the above, possibly with the addition of pus, pin loosening, or increased microbial growth on cultures.

Recognizing that these definitions can be tedious a pin site infection may be defined, for practical purposes, as the presence of any classic signs or symptoms of infection around a pin or wire that requires treatment with antibiotics, pin removal, or debridement [36]. Once determined an infection, various classification systems may be employed to describe the severity of the infection further. Four classes used commonly are in Table 1 [7, 11, 45, 51].

**Table 1:** Four commonly used classification systems to describe pin site infections, as described by Ward, Saleh and Scott, Checketts et al., and Dahl et al.

Classification systems
*Ward (1998)*
Minor—Prolonged drainage, crusting, swelling, and erythema. Considered benign
Major—Resolution requires removal of affected pins
*Saleh and Scott (1992)*
Grade 0—No problems
Grade 1—Responds to local treatment, increased cleaning, and massage
Grade 2—Responds to oral antibiotics
Grade 3—Responds to intravenous antibiotics or pin releases
Grade 4—Responds to removal of the pin
Grade 5—Responds to local surgical curettage
Grade 6—Chronic osteomyelitis
*Checketts*–*Otterburns Grading System (1999)*
Grade 1—Slight erythema, little discharge. Treat with improved local pin care
Grade 2—Erythema, discharge, pain, warmth. Treat with improved local pin care and oral antibiotics
Grade 3—As per grade 2, but no improvement with oral antibiotics. Pins/ex fix can be continued
Grade 4—Severe soft tissue infection involving several pins ± pin loosening. Ex fix must be discontinued
Grade 5—As per grade 4, but with bone involvement visible on radiographs. Ex fix must be discontinued
Grade 6—Major infection occurring after ex fix removal. Treatment requires curettage of pin track
*Dahl Wire and Pin Site Classification and Treatment (1994)*
Grade 0—Normal. Treat with weekly pin care
Grade 1—Inflammed. Daily pin care
Grade 2—Serous drainage. Antibiotics
Grade 3—Purulent discharge. Antibiotics
Grade 4—Osteolysis. Pin removal
Grade 5—Ring sequestrum. Debridement

### Risk factors for acquiring pin site infections

Not all patients are susceptible to pin site infections [15, 17, 48, 50, 57] due to a combination of factors related to patient health and the external fixator. Patient factors associated with a higher risk of pin site infections include increased patient age and intrinsic medical comorbidities [15]. The immune status and consumed medications are expected to influence the risk of infection; examples include diabetes, rheumatoid arthritis and other collagen vascular diseases, and use of steroids. Smoking has been studied extensively and has been shown to increase post-operative complications including wound infection [32]. Although not studied directly in the context of percutaneous pin and wire infection, smoking has been shown to decrease subcutaneous collagen production [22]. Ceasing to smoke preoperatively has been demonstrated to reduce wound-related complications dramatically in patients undergoing primary hip and knee arthroplasty [32]. Anecdotally, we have observed that excessive patient activity leads to increased pin irritation and infection also and that traumatized skin is less resistant to infection.

External fixator parameters affect the risk of pin site infections. Increased duration of pin fixation was associated with a higher rate of pin site infection in a cohort of 27 patients with 178 total pin site infections [17]. This study also suggested that periarticular pin placement was associated with a greater infection rate than diaphyseal placement (1.6 % vs. 4.5 %, *p* < 0.01) [17] possibly due to increased soft tissue motion about joints. Sites with greater soft tissue thickness over bone have been implicated as at higher risk of infection. The infection rate has been reported to be 2.5-fold greater in patients with external fixators performing active correction than those which are not [48]. Skin tension around a pin site has also been associated with greater infection rates [57]. Pin insertion technique may influence the risk of developing a pin site infection: pre-drilling pin sites with a sharp dill bit; meticulous soft tissue handling; inserting pins by hand; and not using a tourniquet to reduce the risk of thermal necrosis to bone and skin may decrease the risk of infection. We have observed also that inadequate fixation may place excessive load on too few fixation points and lead to infection.

### Complications resulting from pin site infections

Although pin site infections are complications, severe problems may follow that could compromise treatment goals and increase patient morbidity [31, 36, 39]. These include pin loosening (with loss of fixation, loss of alignment, frame instability, and, in rare cases, abandoning external fixator treatment [26, 28]), osteomyelitis, joint or fracture site contamination, and increasing pain which limits patient function. Loose pins and wires, when identified, should be removed promptly to prevent progression to osteomyelitis and the pins and wires vital to construct integrity need to be replaced. Osteomyelitis may arise from superficial pin site infections in up to 4 % of cases and represents the most severe consequence of a superficial infection [36]. Timely, meticulous surgical debridement can prevent this from becoming chronic osteomyelitis.

In the presence of a pin site infection, the risk of intramedullary infection is increased from 6 to 70 % in patients undergoing conversion from external to internal fixation (*p* = 0.003) [31]. These observations were based on the treatment of severe open tibia fractures with an intense post-injury inflammatory response and that the conversion from external to internal fixation was immediate without a hardware-free period to treat the pin infections. Furthermore, the half pins used in these patients were mostly 5 mm, self-drilling and not hydroxyapatite (HA)-coated pins—all of which make infection more probable. The conversion from external fixation to internal fixation has become common in limb lengthening and is highlighted by the lengthening and then nailing (LATN) technique [43]. In LATN, there is no contact between the components used for external and internal fixation. The pins and wires are placed peripherally in the proximal tibia and distal to the planned site of the tip of the nail (out of the way of the future IM nail) to prevent the nail from passing through a previous pin site to minimize contamination. Pre-drilling the bone, using 6-mm HA-coated pins, and meticulous technique can make the conversion from external to internal fixation safe. In cases where eccentric pin placement was not used and conversion to internal fixation after prolonged external fixation is needed, we used a specific protocol; the external fixator was removed, the limb was casted, and internal fixation delayed for a minimum of 1 month [44]. With this method, deep infection occurred in 2.5 % of patients and resolved without sequelae after hardware removal and 6 weeks of intravenous antibiotics [44]. Recently, we have used an intramedullary nail that is coated with antibiotic infused bone cement [65].

## Evidence for prevention of pin site infections

Although pin infections are a common complication of external fixation, there is no consensus regarding the optimal measures that should be used to prevent them. The variations in surgeon and nursing preference are, in part, due to limitations and gaps within the current literature. Specific limitations, outlined in Table 2, include a paucity of randomized controlled trials, meta-analyses, and the lack of control groups in many studies. Many studies evaluate different variables (i.e. cleansing solution, dressing type, frequency of cleaning), making it difficult to discern the effect of any one variable in the event of a positive result. Despite these shortcomings, this section reviews the evidence and includes the senior authors’ preferred pin site care protocol.

**Table 2:** Limitations of the current literature posing a barrier to the study of pin site infection preventative strategies

Limitations of the current literature	Implication
Lack of uniform definition/criteria to diagnose and classify severity of pin tract infections	Difficult to study incidence Inaccurate diagnoses complicate interpretation of intervention efficacy
Highly variable control groups between studies (different baseline of prophylactic antibiotics, pin care protocol, etc.)	Difficult to apply study results to an individual practice Difficult to conduct meta-analysis
Within a given study, treatment groups that differ by more than one variable (e.g. changing both the cleansing solution and dressing type)	Impossible to discern effect of individual variables Difficult to apply study results to an individual practice
Few randomized controlled trials	Base clinical practice on low quality, underpowered, potentially biased studies
No consistency in reporting infection rate (per patient vs. per individual pin site)	Difficult to study incidence Difficult to compare studies Difficult to conduct meta-analysis

### Pin design

Attention has been placed on the development of materials and specialized coatings of pins that could potentially reduce rates of infections. Examples include titanium pins and hydroxyapatite (HA) coatings. Titanium is thought to improve the metal–skin interface by inciting a smaller inflammatory response than stainless steel. HA enhances osseointegration of the pin, decreases motion at the interface, and lowers the loosening rate which would, otherwise, be a major contributor to infection.

In a randomized controlled trial of 80 patients (320 pins) with unstable distal radius fractures treated with small AO external fixators, patients had either titanium alloy or stainless steel pins of identical geometry. After an average of 44 days in the external fixator, there were no statistically significant differences in the total pin site infection rate (erythema, cellulitis, drainage), pin loosening, or need for premature fixator removal (*p* > 0.05 for all outcomes). It was concluded that the additional cost of titanium alloy pins was not warranted given that pin site complications were not different compared with stainless steel pins.

In a randomized study of 19 patients (76 pins) undergoing hemicallotasis in the treatment of medial compartment knee osteoarthritis, Magyar et al. [27] demonstrated that HA-coated Orthofix pins had a lower rate of loosening and greater extraction torques than uncoated pins. Similar results were seen in a sheep model in which HA-coated pins had a significantly greater extraction torque and enhanced bony ingrowth (per microscopy) in comparison with titanium-coated and uncoated pins [33]. There is a hypothesis that the HA coating is resistant to bacterial adhesion [3]; in this in vitro study, the adherence of *Staphylococcus epidermidis* to stainless steel screws was significantly lower in the presence of HA coating.

Pommer et al. [39] conducted a similar study but evaluated pin site infection rate, pin removal, and pin extraction torque as the outcomes of interest. A total of 46 patients undergoing segmental bone transport or tibial lengthening were randomized to the use of either standard titanium or HA-coated stainless steel Schanz pins and were followed prospectively for a mean of 38 weeks. The uncoated pins had a 12 % infection rate with 1 extensive intramedullary canal infection, while none of the HA-coated pins became infected. None of the HA-coated pins required removal throughout the study in contrast to 13 % of the uncoated pins. The extraction torque of the HA-coated pins was significantly greater than the uncoated pins (0.43 vs. 0.10 N m, *p* < 0.001).

In the context of a systematic review where the qualifying studies are evaluated for possibilities of confounding or bias, the conclusion was although HA pin coating reduced rates of loosening, there was insufficient evidence to determine whether this brought benefits to rates of deep infection, malunion, or the need for pin removal secondary to infection [62].

Pin geometry and thread design are parameters that has been studied in the context of reducing pin site infection. W-Dahl and Toksvig-Larsen [54] randomized patients undergoing hemicallotasis osteotomy to have either XCaliber (Orthofix) pins with optimized thread and tip design or standard Orthofix pins. In both groups, HA-coated pins were used in the metaphyseal bone, whereas non-coated pins were used in diaphyseal bone. At 7 weeks post-operatively, there were no differences in use of antibiotics (10.5 days with XCaliber, 7.5 days with standard; *p* = 0.16), although the XCaliber group had significantly more pain at rest, pain during activity and greater paracetamol use. The authors concluded that the thread design of the standard pin was adequate in this clinical setting.

Alternative pin coatings, including silver and gold, have been used experimentally to reduce infection. These have been shown to prevent bacterial adhesion or growth, but clinical use is not widespread possibly due to the associated expense and need for further clinical testing.

### Surgical technique

Surgical technique is quoted anecdotally as a means of lowering the incidence of pin infection. The goal is to prevent injury to the bone and soft tissues and subsequent bacterial colonization of necrotic tissue. Intra-operative precautions such as protecting soft tissues with drill sleeves, using sharp drill bits, avoiding thermal necrosis when using power drills, and preventing ischaemic necrosis of skin by implanting wires or pins without excessive skin tension are all measures thought to reduce pin site infections [16, 37, 42]. Although these techniques have not been studied formally, they represent good practice. Taking measures to reduce thermal and mechanical damage of bone during pin insertion are important as these factors have been linked to fibrous tissue formation, pin loosening, and infection [33, 57]. Unicortical placement of pins and wires can generate excessive heat and burn the bone and should be avoided. Tourniquet use during pre-drilling and wire insertion will decrease blood flow to the bone and tissues preventing blood from naturally “cooling” the bone; this may, in turn, result in thermal necrosis of bone. In addition to thermal damage, haematoma formation is associated with greater rates of pin site infection [12].

### Cleansing solutions

Many investigators have attempted to determine the efficacy of different cleansing solutions in reducing the rate of pin site infection. This section will focus on the few randomized studies that aimed to determine the effect of using different cleansing solutions as the main variable.

Egol et al. [15] published a level II randomized controlled trial in which 118 patients undergoing external fixator treatment of unstable displaced distal radial fractures were randomized into one of three groups: (1) no cleansing solution with weekly dry dressing changes, (2) half-strength hydrogen peroxide applied daily, and (3) Biopatch^®^ chlorhexidine-impregnated discs changed weekly. The study was powered to detect a 5 % difference in infection rate, but after a mean follow-up of 5.9 weeks, the study failed to reveal any significant differences in pin site erythema, drainage, cellulitis, antibiotic use, pin removal rate, and pin loosening. It was concluded that sterile dry dressings changed weekly are an adequate and inexpensive choice; the study limitations include unblinded evaluation of infection and dressing changes done by the treating surgeon and of uncertain patient compliance. Henry [20] led a prospective randomized controlled trial evaluating the efficacy of 0.9 % sodium chloride solution versus 70 % isopropyl alcohol in the setting of paediatric leg lengthening and found no significant difference in pin site infection rate (25 and 18 %, respectively). Patterson [37] completed a multicenter randomized controlled trial of 92 patients treated with external fixators. There was no significant difference in pin site infection rate between three different cleansing solutions: (1) 0.9 % saline; (2) half-strength hydrogen peroxide; and (3) soap with water (30, 27, and 45 % incidence of pin site infection, respectively). Despite this, the authors were concluded the combination of half-strength hydrogen peroxide with Xeroform dressings was superior to soap and water cleansing with dry gauze. Despite randomization, the impact of these studies is limited by relative infrequent use of external fixation in favour of volar plating in the distal radius [15], inadequate statistical analysis [20], and inadequate study design without published inclusion criteria [37].

Lethaby et al. [25] published a Cochrane systematic review and meta-analysis that pooled the data of the three negative studies by Egol [15], Henry [20], and Patterson [37]. The meta-analysis considered the outcome of pin site infection rate and was based upon two study groups: 1) patients receiving any type of pin site cleansing solution versus 2) patients receiving no cleansing regimen [25]. These two broad groups were chosen for the meta-analysis because of the heterogeneity between the three studies chosen for the analysis used different control pin site care protocols and tested different dressing types. There was insufficient evidence to suggest that the use of pin site cleansing solutions reduces pin site infection rates (relative risk (RR) 2.30, 95 % confidence interval 0.63–8.33) [25]. Although the authors employed specific inclusion and exclusion criteria, they maintained this conclusion may be difficult to interpret given that the meta-analysis was based upon small, non-blinded, heterogeneous studies at a high risk of bias. Five years after publication of this meta-analysis, an additional Cochrane review drew similar conclusions that there was insufficient evidence to promote any specific strategy of pin site infection prophylaxis [59].

W-Dahl and Toksvig-Larsen conducted a prospective cohort study of 49 patients (196 pins) undergoing tibial osteotomy and external fixation for knee deformity correction in which pin site infection rates were compared at 1, 6, and 10 weeks post-operatively with two different cleansing solutions: (1) chlorhexidine (2 mg/ml) and (2) normal saline (0.9 % sodium chloride) cleansing solutions [52]. It was concluded that the chlorhexidine solution was superior to saline, as the saline group demonstrated more frequent positive pin site cultures (RR 1.7, *p* < 0.0001), more frequent presence of *S. aureus* (RR 3.3, *p* < 0.0001), greater use of antibiotic treatment (22 vs. 9 days, *p* = 0.002), and greater use of pain medications at the time of pin extraction (*p* = 0.03). However, there was no significant difference in infection rates, rates of pin loosening, or incidence of higher grade infections on the Checketts–Otterburns scale. Although this study demonstrates that a chlorhexidine cleansing solution may lead to lower rates of pin site bacterial colonization and decreased use of antibiotics and pain medications as compared with saline, it is limited in that the pin site infection rate was not shown to differ. A more recent cohort study suggested that chlorhexidine cleansing solution leads to decreased pin site infection rates. However, surgical techniques and other aspects of pin site care differed between the two study groups confounding the interpretation of the results [12].

In summary, it is unclear whether cleansing pin sites is necessary to reduce the risk of infection. The ideal pin site cleansing solution is yet to be identified, but there is some evidence that chlorhexidine may be useful to decrease pin site colonization, antibiotic use, and pain.

### Frequency of pin site cleaning

The optimal frequency for pin site care is unclear. One study found that the frequency of pin site cleaning is a factor that could potentially affect pin site infection rates [55]. Fifty patients undergoing tibial osteotomy and external fixation for gonarthrosis were prospectively randomized to receive daily or weekly pin site cleansing with a 0.9 % saline solution. It was concluded that weekly pin site cleaning is appropriate in this patient population, as there was no difference in pin site infection rate (1.5 vs. 1.6 % per pin), infection severity, antibiotic use (42 vs. 53 days), and analgesic use between the daily and weekly cleaning groups, respectively.

### Dressing types

Does the type of pin site dressing influence infection rate? Although many investigators have looked into this, a conclusion on the efficacy of certain dressings alone is not possible because the study groups differ in other aspects of pin site care or surgical technique. Seven randomized trials were identified that were designed to specifically evaluate the efficacy of various dressings in reducing pin site infection rate [1, 4, 15, 18, 37, 60, 61]. Lee and colleagues [60] determined that pin infection rate was lower in patients undergoing limb lengthening procedures with a polyhexamethylene biguanide dressing as compared to dry gauze (1.0 versus 4.5 %, respectively); however, no deep infections occurred in either group. Ogbemudia et al. [61] concluded that a 1 % silver sulphadiazine dressing reduced pin track infection rates in a population comprised mostly of trauma patients (1.9 % of pin sites, as compared to 14.2 % with dry gauze). However, Yuenyongviwat and Tangtrakulwanich [1] found no differences between these dressing types in the setting of tibial external fixators used for trauma (46.7 % of patients with pin track infections with silver sulphadiazine dressings versus 40.0 % with dry gauze).

In the remaining four randomized trials, no dressing type was shown to be superior [4, 15, 18, 37]. Egol et al. [15] studied dry dressings and the chlorhexidine-impregnated patch (Biopatch^®^) in adults with unstable displaced distal radius fractures. Grant et al. [18] compared 10 % betadine gauze with white paraffin ointment in adult patients, although specific inclusion criteria were not provided. Camilo et al. [4] studied polyvinylpyrrolidone–iodine-soaked gauze versus dry gauze in the setting of Ilizarov external fixators. Patterson [37] did not list specific inclusion criteria, but compared dry gauze changed twice daily, dry gauze changed only when appearing soiled, and Xeroform dressings changed twice daily. An attempt to pool the results of these four studies in the form of a meta-analysis was deemed to be unfeasible due to the extent of heterogeneity between the studies [25].

### Showering

While many orthopaedic surgeons have guidance on when patients may shower or bathe post-operatively with the intent of reducing the risk of pin site infection, there is little direction from the literature. One case series suggested that showering after post-operative day (POD) 5 is not only acceptable, but could be used successfully as the only means of pin site care [17]. The authors followed 27 children with tibial external fixators for deformity correction or limb lengthening for a mean of 22.4 weeks, using daily showering as the only method of pin site care after dressings were removed on POD 5. The authors concluded that daily showering was adequate as the sole measure for pin site care as all infections resolved on oral antibiotics and no Dahl grade 3–5 infections occurred. However, the study lacked a control group, and approximately 75 % of the pin sites (178 total infections out of 136 half pins and 76 wires) became infected at some point during the study.

There are a small number of studies that seek to determine when surgical wounds may be exposed to showering. A cohort study of 192 patients undergoing thoracolumbar spinal operations showed no difference in wound infection rates with early showering (POD 2 for lumbar microdiscectomy, POD 5 for other operations; 2 % wound complication rate) versus a control group (showering on POD 10–14 after staple removal; 4 % wound complication rate) [6]. These patients were advised to dry the wound and apply sterile gauze after showering. In this setting, the authors concluded that early showering was not harmful. Two prospective randomized trials demonstrated no difference in infection rate with early showering in the settings of open hernia repair and varicose vein surgeries [34, 41]. It is unclear whether these results can be extrapolated to pin sites as these are not closed wounds. Further research in this area would be helpful.

### Prophylactic antibiotics

Oral antibiotics are often prescribed for prophylaxis against pin site infection, but evidence-based guidelines are lacking [36]. To address this issue, W-Dahl published a cohort study investigating the effect of a single dose versus 3 days of prophylactic antibiotics on the pin site infection rate in 106 patients with elective tibial osteotomy and external fixation for knee deformity [53]. In this study, patients received either a single preoperative dose of IV cloxacillin (2 g) given 20–30 min prior to incision, or a preoperative dose of IV cloxacillin (2 g) followed by two IV doses over the first 24 h followed by oral prophylaxis (flucloxacillin 1 g × 3) for an additional 2 days. The study found no significant difference in use of treatment antibiotics, pin loosening, positive pin site cultures, the presence of *S. aureus*, or distribution of infection grade at weeks 1, 6, and 10 post-operatively. Therefore, it was concluded that a single preoperative dose of prophylactic antibiotics is adequate in this clinical setting.

A study by Magyar et al. [26] supported use of oral prophylactic antibiotics for 2 weeks post-operatively in their series of 308 consecutive patients treated with open-wedge osteotomy by hemicallotasis for osteoarthritis of the knee. Their data revealed that the infection rate was approximately 80 % when 0–3 days of prophylactic antibiotics were given and nearer 40 % in those who received 11–17 days of prophylactic antibiotics. The infection rate did not decrease further when antibiotic prophylaxis was extended beyond this time period. However, the authors did not provide statistical analysis or sample sizes, making it difficult to interpret these data.

In addition to oral prophylactic antibiotics, local administration of antibiotics has been hypothesized to reduce rates of pin site infection [35]. A total of 60 patients admitted for a variety of orthopaedic conditions (intertrochanteric fracture, femoral neck or diaphyseal fracture, or hip and knee deformity) were designated to receive either 250 mg cephazolin injections along the pin insertion site or no antibiotic prior to placement of a proximal tibia Steinmann pin for skeletal traction. After an average of 25 days of traction in the antibiotic group and 29 days in the control group, pin site infection rates were 3 and 30 %, respectively (*p* = 0.003). Therefore, the authors concluded that the local antibiotic administration reduced pin site infection rates, likely by reducing local bacterial flora.

Chou et al. [9] reported that topical antimicrobial application to the metal–skin interface reduced the pin site infection rate in a rabbit model. In this study, 37 rabbits were randomized to one of three groups: (1) titanium alloy implant with no antimicrobial, (2) titanium alloy implant with topical antimicrobial (1 % pexiganan acetate) applied to the skin–metal interface daily, and (3) a porous tantalum implant with no antimicrobial. A 75 % reduction in pin site infections was achieved with the titanium–pexiganan group in comparison with the titanium control group (*p* = 0.019). However, there was no difference in infection rate between the titanium control and tantalum groups (*p* = 0.230).

### Other factors

In addition to the factors discussed, there are other aspects of pin site care or operative technique that may affect infection rates. There are no controlled studies that address whether to use sterile or non-sterile technique when administering pin site care. There is an uncertainty over the effects of pin site massage to relieve skin tension, whether dressings are advantageous (versus no dressing) and whether to remove pin site eschar.

### Our protocol

Over 200 new external fixators are placed annually at the senior authors’ institution; the management of pin sites and pin site infections is an essential part of daily clinical practice. The prevention of infection protocol includes the following: intravenous antibiotics are given prior to skin incision and continued for 24 post-operatively; no tourniquet is used; and pins are inserted after pre-drilling with sharp drills using sleeves to protect soft tissues. Electrocautery is avoided at pin sites to prevent tissue necrosis. HA-coated tapered pins with cortical threads are used exclusively. For most applications, 6.0-mm-diameter pins are used; however, 4.5-mm pins are used in adult foot and forearm cases and for paediatric patients. Pin diameter is ensured to be <33 % of the bone diameter. Pin site care starts on POD 2. Pin care is done once daily by cleaning each pin site with a new sterile cotton-tipped applicator that has been soaked in a solution of 1:1 hydrogen peroxide and normal saline. Dry sterile gauze is wrapped around each pin site. Daily showering is encouraged on POD 4 where the patients remove the dressings, allowing water to rinse the frame and use of an antibacterial liquid soap. The leg and frame are patted dry with a clean towel. Patients are allowed to swim in a chlorinated pool after 4 weeks. Most patients are allowed weight bearing as tolerated. Over time, some patients develop a non-infectious dermatitis for which we have learned that further dilution of the hydrogen peroxide solution is helpful. For example, instructions would be changed to use 1 part hydrogen peroxide and 3 parts normal saline.

When a patient presents with a pin site reaction, they are encouraged to start twice daily pin care for that pin, reduce activity and weight bearing, and start oral antibiotics if these measures fail or if symptoms worsen. If a patient presents with a pin site infection, they are started on oral antibiotics and instructed to start twice daily pin site care for that pin. A reduction of weight-bearing activity in conjunction with elevation of the limb (if swelling is present) is recommended. If the infection does not resolve completely, pin cultures are taken and a second antibiotic is added empirically. Culture-specific antibiotics are then started when the results become available. For persistent infections, radiographs are obtained and evaluated for pin loosening. If half-pin loosening is not clear from the radiographs, the pin is then disconnected from the frame and tested. Loose pins are removed immediately in the office. Persistently infected tensioned wires are also removed in the clinic. When vital pins or wires are removed, the patient is brought to the operating room for frame modification urgently. At that time, the pin site is debrided and if there is accompanying cellulitis, the patient admitted for IV antibiotics and elevation. Patients who have had deep infections (loose, infected pin with lucencies on X-ray) are monitored for signs of osteomyelitis. Typically, removal of the foreign body (the half pin) is enough to cure the infection. When in doubt, an MRI (after frame removal) is obtained to evaluate the need for repeat debridement. After treatment with the external fixator is complete and at the time of frame removal, all pin sites are debrided. Since HA pins have been used routinely, loose pins are very rare at the time of frame removal and most pins are still fixed securely in the bone.

## Future directions

Pin site infections remain a common clinical problem in patients treated with external fixators. Future research in this area holds promise in elucidating additional effective preventative measures. Given the limitations of the current literature, well-designed clinical trials will be instrumental in assisting clinicians choose optimal pin site care regimens, pin designs, and operative techniques when working with patients with percutaneous orthopaedic pins and wires. Techniques which reduce intramedullary nail contamination for patients previously treated with external fixation can minimize deep infection, including with the LATN technique [43] or by avoiding penetration of the canal altogether through use of monocortical screws held by a specialized clamp in lieu of traditional pins and wires. Consideration of these techniques for surgical applications beyond the tibia may worthwhile.

Continued focus on materials development and novel methods of prophylactic antibiotic administration may provide insights that can be tested further in human clinical trials. Specifically, efforts are being made to produce an improved pin coating that has antimicrobial properties. This would have implications for external fixation but would revolutionize internal fixation for use over a site of previous pin use and for routine use as a prophylactic measure against infection. The findings of Chou et al. [9] are intriguing; topical pexiganan acetate applied to pin sites resulted in a 75 % decrease in pin site infections in a rabbit model. DeJong et al. [13] found that the pin site infection rate was significantly decreased by adding a coating of chlorhexidine, hydroxyapatite, and lipid to titanium and stainless steel pins implanted into goat tibiae. The coated pins had a 4.2 % infection rate and 12.5 % colonization rate at 14 days after inoculating the pin sites with *S. aureu*s, in contrast to the 100 % infection rate in the uncoated pins (*p* < 0.01). Although the extraction torque of the coated pins decreased over the 14-day study period, it was superior to the uncoated pins. These results suggest that this coating may be a viable option to reduce the risk of infection while maintaining a stable interface with bone. In an in vitro study by Chen et al. [8], a silver-containing HA pin coating was shown to decrease *S. aureus* and *S. epidermidis* adhesion in comparison with plain titanium pins without increased cytotoxicity to precursor osteoblast cells. The prevention measures tested in the Chou [9], DeJong [13], and Chen [8] studies, which seem promising in vitro or in animal models, would need to be shown to be effective in patients.

Puckett et al. [40] have taken a unique approach to pin design. The break in the protective skin layer at the skin–metal interface is thought to facilitate the passage of bacteria and formation of pin site infections. Therefore, it was hypothesized that the infection rate may improve if a continuous skin–metal interface is achieved. In order to accomplish the improved interface, a nano-roughened titanium pin material was developed that promotes improved keratinocyte adhesion. Follow-up studies based on this technology are pending.

## Summary and conclusions

There is no consensus on the precise definition of a pin site infection, but this frequent complication of external fixation is a cause of considerable cost and patient morbidity. Patients with advanced age, multiple medical comorbidities, a prolonged duration of treatment in the external fixator, and those undergoing active correction are at an increased risk of pin site infections which may lead to additional complications such as osteomyelitis, pin loosening, loss of alignment, or premature removal of the external fixator.

The literature is limited with regard to prevention of pin site infection. A small number of studies have been published that guide the orthopaedic surgeon to choosing strategies to reduce the risk of pin site infections: hydroxyapatite pin coatings improve osseointegration, extraction torque, and pin site infection during bone transport or tibial lengthening; meticulous operative technique is an important factor—soft tissue disruption and thermal damage should be minimized when inserting pins or wires—and care should be taken to reduce the skin tension at pin or wire sites.

There is no strong evidence to guide choice of dressing type, cleansing regimen, or other aspects of pin site care. There is suggestion that chlorhexidine may be superior to saline as a pin site cleansing solution and that daily cleansing with saline is not superior to weekly cleansing. With regard to pin site dressings, there is suggestion that polyhexamethylene biguanide dressings, and possibly silver sulphadiazine dressings, may reduce pin track infection rates. However, there are several other trials showing the effect of dressing type to be negative and the question remains as to whether post-operative pin site dressings are important. With regard to pin site care, commencement of dressing changes on POD 2–3 may be convenient, as the drainage associated with pin site reaction normally decreases by this time. Clinicians should use personal judgement and experience until better evidence is available and, especially in the light of weak evidence, should consider the cost–benefit ratio of any pin site care regimen used.

The optimal regimen and time course are yet to be determined for prophylactic antibiotics. There is some evidence that 3 days of prophylactic antibiotics is not superior to one preoperative dose in the setting of elective tibial osteotomy and external fixation for knee deformity. In patients undergoing treatment with opening-wedge osteotomy by hemicallotasis for osteoarthritis of the knee, 2 weeks of prophylactic antibiotics was determined to be optimal, although the study was limited by a lack of statistical analysis. As for local prophylactic antimicrobials, there is some clinical and laboratory evidence that administering them reduces the incidence of pin site infections, although this method is not a standard of practice currently. Until more evidence is available, the choice of prophylactic antibiotic regimen should be guided by the clinician’s experience for the particular orthopaedic operation, by patient comorbidities and a past history of infection.

As many questions remain over how to reduce the risk of pin site infections effectively in patients treated with percutaneous orthopaedic pins and wires, an important measure that can be taken at this point in time is to teach patients how to recognize the signs and symptoms of pin site infection promptly so they may seek treatment as soon as possible. Specifically, pain at a pin site may precede infection at which point oral antibiotic treatment may be initiated [17].

Surgeons and nursing staff should adopt a uniform pin care protocol that works for their patients and that can be taught to everyone involved in that patient’s care. Using a consistent protocol will help to ensure that the patient is not getting different information from different members of the healthcare team, a common problem that can lead to confusion and loss of confidence. Providing patients with a handout describing the pin site care protocol is an effective way to communicate to home nursing and family members that are involved in the pin site care. Audits of the protocol with a review of the latest studies on pin infection and prevention will allow for updating the protocol and delivering high-quality care.
